# Supplementary material for: A population-based cohort study of obesity, ethnicity and COVID-19 mortality in 12.6 million adults in England
Source: Nat Commun. 2022 Feb 2;13:624. doi: 10.1038/s41467-022-28248-1 (PMC8810846; doi:10.1038/s41467-022-28248-1)
Supplement: Supplementary file 1 — Supplementary Information [file 41467_2022_28248_MOESM1_ESM.pdf]

A population-based cohort study of obesity, ethnicity and COVID-19 mortality in 12.6 million adults in England

Supplementary Information

Contents

Supplementary Table 1: Population characteristics, stratified by ethnicity. 1

Supplementary Table 2. Comparison of demographic and medical characteristics by those with and without valid body mass index data. 6

Supplementary Table 3: Statistics showing predictive discrimination for missing data. 10

Supplementary Figure 1: BMI equivalency values for Class II and Class III obesity thresholds across ethnic groups. 11

Supplementary Figure 2: Association of BMI with COVID-19 mortality by ethnicity, adjusted for clinical factors. 12

Supplementary Figure 3. Association of BMI with COVID-19 mortality by ethnicity in women. 13

Supplementary Figure 4: Association of BMI with COVID-19 mortality by ethnicity in men. 14

Supplementary Figure 5: Association of BMI with COVID-19 mortality by ethnicity: individuals 70 years or older. 15

Supplementary Figure 6: Association of BMI with COVID-19 by ethnicity: individuals younger than 70 years. 16

Supplementary Figure 7: Association of BMI with COVID-19 mortality across ten ethnic categories. 17

Supplementary Figure 8: Flow diagram of cohort. 18

Supplementary Figure 9: Percentage of missing BMI data in each ethnic group by region. 19

Supplementary Table 1: Population characteristics, stratified by ethnicity.

		White (*n* = 11,074,708)	Black (*n* = 416,542)	South Asian (*n* = 621,691)	Other (*n* = 478,196)
Age (yr) (mean [SD])		61.9 (13.4)	56.4 (11.7)	55.7 (12.4)	55.3 (11.6)
Sex (%)	Male	5,139,079 (46.4)	177,981 (42.7)	304,916 (49.1)	215,736 (45.1)
	Female	5,935,629 (53.6)	238,561 (57.3)	316,775 (51.0)	262,460 (54.9)
Body Mass Index (kg/m^2) (mean [SD])		27.9 (5.7)	29.3 (5.9)	27.2 (5.0)	26.7 (5.2)
Region (%)	East	527,620 (4.8)	7,958 (1.9)	16,732 (2.7)	15,908 (3.3)
	East Midlands	305,002 (2.8)	3,706 (0.9)	9,329 (1.5)	5,242 (1.1)
	London	1,404,653 (12.7)	282,743 (67.9)	279,403 (44.9)	240,467 (50.3)
	North East	477,650 (4.3)	1,789 (0.4)	4,940 (0.8)	6,999 (1.5)
	North West	2,474,713 (22.4)	27,346 (6.6)	94,299 (15.2)	53,092 (11.1)
	South East	2,639,897 (23.8)	34,334 (8.2)	75,532 (12.2)	83,377 (17.4)
	South West	1,015,274 (9.2)	9,868 (2.4)	9,654 (1.6)	16,072 (3.4)
	West Midlands	1,734,175 (15.7)	43,509 (10.5)	124,469 (20.0)	48,894 (10.2)
	Yorkshire and the Humber	495,724 (4.5)	5,289 (1.3)	7,333 (1.2)	8,145 (1.7)
Urban Rural classification (%)	Rural hamlets and isolated dwellings	434,588 (3.9)	899 (0.2)	1,774 (0.29)	3,840 (0.8)
	Rural hamlets and isolated dwellings in a sparse setting	38,604 (0.4)	29 (0.0)	36 (0.0)	206 (0.0)
	Rural town and fringe	954,956 (8.2)	2,801 (0.7)	5,418 (0.9)	10,626 (2.2)
	Rural town and fringe in a sparse setting	39,825 (0.4)	59 (0.0)	86 (0.0)	342 (0.1)
	Rural village	679,768 (6.1)	1,445 (0.6)	2,781 (0.5)	6,134 (1.3)
	Rural village in a sparse setting	44,749 (0.4)	36 (0.0)	50 (0.0)	258 (0.1)
	Urban city and town	4,633,331 (41.8)	54,516 (13.1)	147,247 (23.7)	116,791 (24.4)
	Urban city and town in a sparse setting	23,073 (0.2)	30 (0.0)	91 (0.0)	204 (0.0)
	Urban major conurbation	4,041,153 (36.5)	352,179 (84.6)	458,337 (73.7)	334,828 (70.0)
	Urban minor conurbation	184,661 (1.7)	4,548 (1.1)	5,871 (0.9)	4,967 (1.0)
Population density (people per square kilometre)(mean [SD])		3,873.7 (3,929.8)	9,927.1 (6,316.8)	8,111.8 (5,966.4)	7,647.6 (6,045.6)

Household deprivation (%)	Not deprived in any dimension	5,398,007 (48.7)	150,596 (36.2)	224,081 (36.0)	196,694 (41.1)
	Deprived in 1 dimension	3,407,802 (30.8)	147,433 (35.4)	208,303 (33.5)	158,813 (33.2)
	Deprived in 2 dimensions	1,629,446 (14.7)	83,187 (20.0)	133,715 (21.5)	84,671 (17.7)
	Deprived in 3 dimensions	462,202 (4.2)	27,292 (6.6)	48,372 (7.8)	30,195 (6.3)
	Deprived in 4 dimensions	42,322 (0.4)	3,585 (0.9)	5,033 (0.8)	4,384 (0.9)
	No code	134,929 (1.2)	4,449 (1.1)	2,187 (0.4)	3,439 (0.7)
Material deprivation (IMD decile) (%)	1 (most deprived)	825,760 (7.5)	88,063 (21.1)	117,028 (18.8)	58,461 (12.2)
	2	888,014 (8.0)	103,716 (24.9)	100,441 (16.2)	69,802 (14.6)
	3	963,592 (8.7)	78,278 (18.8)	79,563 (12.8)	65,706 (13.7)
	4	1,020,432 (9.2)	48,339 (11.6)	68,582 (11.0)	54,962 (11.49)
	5	1,096,988 (9.9)	33,198 (8.0)	61,471 (9.9)	47,924 (10.0)
	6	1,141,078 (10.3)	21,532 (5.2)	53,983 (8.7)	43,776 (9.2)
	7	1,215,851 (11.0)	14,015 (3.4)	39,296 (6.3)	36,208 (7.6)
	8	1,258,087 (11.4)	11,301 (2.7)	34,596 (5.6)	34,084 (7.1)
	9	1,288,482 (11.6)	9,923 (2.4)	32,394 (5.2)	33,809 (7.1)
	10 (least deprived)	1,376,424 (12.4)	8,177 (2.0)	34,337 (5.5)	33,464 (7.0)
Approximate social grade (%)	AB	2,668,369 (24.1)	71,954 (17.3)	160,894 (25.9)	118,489 (24.8)
	C1	3,296,637 (29.8)	131,599 (31.6)	153,071 (24.6)	134,981 (28.2)
	C2	2,402,096 (21.7)	76,811 (18.4)	87,409 (14.1)	79,408 (16.6)
	D	2,343,816 (21.2)	98,784 (23.7)	182,771 (29.4)	112,265 (23.5)
	E	320,788 (2.9)	34,343 (8.2)	36,252 (5.8)	30,694 (6.4)
	No code	43,002 (0.4)	3,051 (0.7)	1,294 (0.2)	2,359 (0.5)
Highest educational attainment (%)	No qualifications	2,501,606 (22.6)	66,187 (15.9)	157,047 (25.3)	91,730 (19.18)
	1-4 GCSE/O-levels	1,523,484 (13.8)	53,064 (12.7)	68,560 (11.0)	45,607 (9.5)
	5+ GCSE/O-levels	1,583,286 (14.3)	55,961 (13.4)	49,393 (7.9)	43,763 (9.2)
	Apprenticeship	467,991 (4.2)	5,594 (1.3)	3,795 (0.6)	4,005 (0.8)
	2+ A Levels or equivalent	1,144,859 (10.3)	39,687 (9.5)	36,337 (5.8)	34,826 (7.3)
	Degree or above	3,338,014 (30.1)	155,420 (37.3)	208,981 (33.6)	179,098 (37.5)
	Other	515,468 (4.7)	40,629 (9.8)	97,578 (15.7)	79,167 (16.5)
Household tenancy (%)	Owned: owned outright	3,880,716 (35.0)	42,051 (10.1)	167,958 (27.0)	86,111 (18.0)
	Owned: owned with a mortgage or loan	4,582,819 (41.4)	129,629 (31.1)	286,544 (46.1)	185,759 (38.9)
	Shared ownership	66,361 (0.6)	6,149 (1.5)	2,953 (0.5)	5,203 (1.1)

	Social rented from council	699,678 (6.3)	86,590 (20.8)	37,233 (6.0)	45,814 (9.6)
	Other social rented	686,324 (6.2)	71,740 (17.2)	33,825 (5.4)	40,654 (8.5)
	Private rented	1,024,833 (9.3)	72,820 (17.5)	84,360 (13.6)	105,581 (22.1)
	Living rent free	90,975 (0.8)	4,512 (1.1)	7,524 (1.2)	6,715 (1.4)
	No code required	43,002 (0.39)	3,051 (0.7)	1,294 (0.2)	2,359 (0.5)
Type of accommodation (%)	Detached	3,127,510 (28.2)	28,945 (7.0)	113,196 (18.2)	77,268 (16.2)
	Semi-detached	3,867,398 (34.9)	89,608 (21.5)	195,917 (31.5)	127,666 (26.7)
	Terraced	2,601,304 (23.5)	115,552 (27.7)	202,687 (32.6)	125,626 (26.3)
	Flat (purpose built)	965,900 (8.7)	148,382 (35.6)	86,771 (14.0	110,458 (23.1)
	Flat (converted)	277,700 (2.5)	26,215 (6.3)	13,453 (2.1	24,345 (5.1)
	Flat (commercial building)	60,018 (0.5)	3,242 (0.8)	7,315 (1.1	9,006 (1.9)
	Other	39,949 (0.36)	149 (0.4)	165 (0.0)	388 (0.1)
	No code	134,929 (1.2)	4,449 (1.1)	2,187 (0.36	3,439 (0.7)
Household size (%)	1-2	7,289,085 (65.8)	223,481 (53.7)	176,472 (28.4)	218,994 (45.8)
	3-4	3,333,717 (30.1)	151,558 (36.4)	298,681 (48.0)	201,504 (42.1)
	5-6	300,440 (2.7)	33,659 (8.1)	114,909 (18.5)	46,748 (9.8)
	7+	16,537 (0.2)	3,395 (0.8)	29,442 (4.7)	7,511 (1.6)
	Missing	134,929 (1.2)	4,449 (1.1)	2,187 (0.4)	3,439 (0.7)
Multigenerational household (%)	No	9,758,322 (88.1)	356,325 (85.5)	452,848 (72.8)	395,678 (82.7)
	Yes	1,181,457 (10.7)	55,768 (13.4)	166,656 (26.8)	79,079 (16.5)
	No code	134,929 (1.2)	4,449 (1.1)	2,187 (0.4)	3,439 (0.7)
Household with children (%)	Yes	1,941,377 (17.5)	133,773 (32.1)	262,010 (42.1)	160,332 (33.5)
Overcrowded (%)	Yes	532,653 (4.8)	113,070 (27.1)	128,682 (20.7)	101,066 (21.1)
Key worker type (%)	Education and childcare	746,622 (6.7)	19,715 (4.7)	26,698 (4.3)	18,542 (3.9)
	Food and necessary goods	65,869 (0.6)	1,756 (0.4)	5,906 (0.95)	2,678 (0.6)
	Health and social care	797,565 (7.2)	64,023 (15.4)	41,345 (6.65)	49,803 (10.4)
	Key public services	185,020 (1.67)	7,181 (1.7)	6,891 (1.1)	6,309 (1.3)
	National and Local Government	100,392 (0.9)	4,625 (1.1)	4,662 (0.8)	2,792 (0.6)
	Not keyworker	8,739,732 (78.9)	307,134 (73.7)	515,151 (82.9)	385,328 (80.6)
	Public safety and national security	158,024 (1.4)	3,860 (0.9)	2,879 (0.5)	3,435 (0.7)
	Transport	129,584 (1.2)	4,571 (1.1)	5,342 (0.9)	3,444 (0.7)
	Utilities and communication	151,900 (1.4)	3,677 (0.9)	12,817 (2.1)	5,865 (1.2)

Key worker in household (%)	No code	134,929 (1.2)	4,449 (1.1)	2,187 (0.4)	3,439 (0.7)
	No	7,170,389 (64.8)	247,815 (59.5)	405,434 (65.2)	318,454 (66.6)
	Yes	3,769,390 (34.0)	164,278 (39.4)	214,070 (34.4)	156,303 (32.7)
Proximity to others* (mean [SD])		58.41 (18.9)	58.95 (25.1)	51.53 (28.1)	56.14 (26.0)
Exposure to disease* (mean [SD])		18.98 (20.7)	26.7 (26.67)	17.3 (22.4)	21.3 (24.7)
Chronic Kidney disease (%)	None, CKD 1-2	10,775,193 (97.3)	405,526 (97.4)	607,621 (97.7)	471,354 (98.6)
	CKD 3	262,284 (2.37)	8,764 (2.1)	11,140 (1.8)	5,586 (1.17)
	CKD 4	26,660 (0.2)	1,237 (0.3)	1,674 (0.3)	659 (0.1)
	CKD 5	10,571 (0.1)	1,015 (0.2)	1,256 (0.2)	597 (0.1)
Learning disability (%)	None	10,935,617 (98.7)	412,954 (99.1)	616,307 (99.1)	474,373 (99.2)
	Learning disability	135,253 (1.2)	3,478 (0.8)	5,244 (0.8)	3,703 (0.8)
	Down's syndrome	3,838 (0.0)	110 (0.0)	140 (0.0)	120 (0.0)
Cancer and immunosuppression (%)	Blood cancer	152,888 (1.9)	3,907 (0.9)	5,236 (0.8)	4,033 (0.8)
	Respiratory cancer	4,666 (0.0)	239 (0.06)	146 (0.0)	112 (0.0)
	Taking immunosuppressants	3,244 (0.0)	62 (0.0)	133 (0.0)	62 (0.0)
	Taking anti-leukotriene or long acting beta2-agonists	1,006,065 (9.08)	21,129 (5.1)	47,665 (7.7)	29,058 (6.1)
	Taking oral steroids in the last 6 months	188,513 (1.7)	3,331 (0.8)	6,555 (1.1)	3,724 (0.8)
Other comorbidities (%)	Cerebral Palsy	1,738 (0.0)	53 (0.0)	102 (0.0)	46 (0.0)
	Asthma	1,355,348 (12.2)	43,649 (10.5)	80,742 (13.0)	54,625 (11.4)
	Atrial Fibrillation	567,932 (5.1)	5,778 (1.4)	8,641 (1.4)	7,682 (1.6)
	Coronary heart disease	756,198 (6.3)	11,536 (2.8)	50,263 (8.1)	21,978 (4.6)
	COPD	544,244 (4.9)	5,060 (1.2)	9,860 (1.6)	8,026 (1.7)
	Cystic fibrosis or bronchiectasis or alveolitis	179,952 (1.6)	4,532 (1.1)	6,336 (1.0)	4,350 (0.9)
	Dementia	204,040 (1.8)	5,910 (1.4)	6,231 (1.0)	3,860 (0.8)
	Diabetes	1,245,180 (11.2)	82,220 (19.7)	174,601 (28.08)	82,897 (17.3)
	Epilepsy	138,698 (1.3)	3,674 (0.9)	4,917 (0.8)	3,831 (0.8)
	Heart failure	267,535 (2.4)	6,447 (1.6)	10,927 (1.8)	5,251 (1.1)

	Liver cirrhosis	39,354 (0.3)	915 (0.2)	1,832 (0.3)	1,414 (0.3)
	Neurological disease	12,318 (0.1)	368 (0.09)	535 (0.1)	380 (0.1)
	Parkinson's disease	57,315 (0.5)	1,076 (0.26)	2,163 (0.4)	1,339 (0.3)
	Peripheral vascular disease	155,578 (1.4)	2,697 (0.65)	4,149 (0.7)	2,379 (0.5)
	Fracture of hip, wrist, spine or humerus	17,341 (0.2)	81 (0.0)	406 (0.1)	203 (0.0)
	Rheumatoid arthritis or SLE	146,556 (1.3)	4,244 (1.0)	9,363 (1.5)	4,949 (1.0)
	Severe mental illness	2,202,390 (19.9)	56,330 (13.5)	80,841 (13.0)	73,540 (15.4)
	Solid organ transplant	1,251 (0.0)	105 (0.0)	115 (0.0)	56 (0.0)
	Stroke or TIA	445,678 (4.0)	11,534 (2.8)	16,798 (2.7)	9,285 (1.9)
	Thrombosis or pulmonary embolus	2,904 (0.0)	27 (0.0)	179 (0.0)	62 (0.0)

Data as number (column %) or mean (SD).

* = score from 0 (no exposure) to 100 (maximum exposure).

Supplementary Table 2. Comparison of demographic and medical characteristics by those with and without valid body mass index data.

Variable	Value	Cases without BMI	Cases with BMI
Ethnicity (%)	Black	209,977 (1.8)	416,542 (3.3)
	Other	319,432 (2.8)	478,196 (3.8)
	South Asian	442,014 (3.9)	621,691 (4.9)
	White	10,464,547 (91.5)	11,074,708 (88.0)
Age (yr) (mean [SD])		60.85 (13.3)	61.16 (13.4)
Sex (%)	Male	5,506,657 (48.2)	5,837,712 (46.4)
	Female	5,929,313 (51.9)	6,753,425 (53.7)
Region (%)	East	2,245,903 (19.6)	568,218 (4.5)
	East Midlands	1,856,050 (16.2)	323,279 (2.6)
	London	714,638 (6.3)	2,207,266 (17.5)
	North East	731,420 (6.4)	491,378 (3.9)
	North West	533,380 (4.7)	2,649,450 (21.0)
	South East	1,284,037 (11.2)	2,833,140 (22.5)
	South West	1,581,588 (13.8)	1,050,868 (8.4)
	West Midlands	564,979 (4.9)	1,951,047 (15.5)
	Yorkshire and the Humber	1,923,975 (16.8)	516,491 (4.1)
Urban Rural classification (%)	Rural hamlets and isolated dwellings	415,515 (3.6)	441,101 (3.5)
	Rural hamlets and isolated dwellings in a sparse setting	38,287 (0.3)	38,875 (0.3)
	Rural town and fringe	1,280,199 (11.2)	973,801 (7.8)
	Rural town and fringe in a sparse setting	55,457 (0.5)	40,312 (0.3)
	Rural village	783,065 (6.9)	690,128 (5.48)
	Rural village in a sparse setting	42,543 (0.4)	45,093 (0.4)
	Urban city and town	5,594,085 (48.9)	4,951,885 (39.3)
	Urban city and town in a sparse setting	21,287 (0.2)	23,398 (0.2)
	Urban major conurbation	2,551,576 (22.3)	5,186,497 (41.2)
	Urban minor conurbation	653,956 (5.7)	200,047 (1.6)
Population density (mean [SD])		3,668.58 (3,936.8)	4,426.54 (4,514.8)
Household deprivation (%)	Not deprived in any dimension	5,485,531 (48.0)	5,969,378 (47.4)
	Deprived in 1 dimension	3,574,785 (31.3)	3,922,351 (31.2)
	Deprived in 2 dimensions	1,714,038 (15.0)	1,931,019 (15.3)
	Deprived in 3 dimensions	482,235 (4.2)	568,061 (4.5)
	Deprived in 4 dimensions	44,150 (0.4)	55,324 (0.4)
	No code	135,231 (1.2)	145,004 (1.2)

IMD decile (%)	1 (most deprived)	862,777 (7.5)	1,089,312 (8.7)
	2	902,810 (7.9)	1,161,973 (9.2)
	3	1,008,728 (8.8)	1,187,139 (9.4)
	4	1,112,875 (9.7)	1,192,315 (9.5)
	5	1,188,222 (10.4)	1,239,581 (9.9)
	6	1,252,507 (11.0)	1,260,369 (10.0)
	7	1,290,824 (11.3)	1,305,370 (10.4)
	8	1,296,152 (11.3)	1,338,068 (10.6)
	9	1,299,601 (11.4)	1,364,608 (10.8)
	10 (least deprived)	1,221,474 (10.7)	1,452,402 (11.5)
Approximate social grade (%)	AB	2,551,383 (22.3)	3,019,706 (24.0)
	C1	3,308,971 (28.9)	3,716,288 (29.5)
	C2	2,571,883 (22.5)	2,645,724 (21.0)
	D	2,620,394 (22.9)	2,737,636 (21.7)
	E	340,823 (3.0)	422,077 (3.4)
	No code	42,516 (0.4)	49,706 (0.4)
Highest educational attainment (%)	No qualifications	2,598,176 (22.7)	2,816,570 (22.4)
	1-4 GCSE/O-levels	1,632,648 (14.3)	1,690,715 (13.4)
	5+ GCSE/O-levels	1,647,767 (14.4)	1,732,403 (13.8)
	Apprenticeship	487,068 (4.3)	481,385 (3.8)
	2+ A Levels or equivalent	1,179,788 (10.3)	1,255,709 (10.0)
	Degree or above	3,262,677 (28.6)	3,881,513 (30.8)
	Other	627,846 (5.5)	732,842 (5.8)
Household tenancy (%)	Owned: owned outright	3,805,386 (33.3)	4,176,836 (33.2)
	Owned: owned with a mortgage or loan	4,813,630 (42.1)	5,184,751 (41.2)
	Shared ownership	69,778 (0.6)	80,666 (0.6)
	Social rented from council	817,907 (7.2)	869,315 (6.9)
	Other social rented	618,705 (5.4)	832,543 (6.6)
	Private rented	1,165,317 (10.2)	1,287,594 (10.2)
	Living rent free	102,731 (0.9)	109,726 (0.9)
	No code required	42,516 (0.4)	49,706 (0.4)
Type of accommodation (%)	Detached	3,451,424 (30.18)	3,346,919 (26.6)
	Semi-detached	3,936,483 (34.4)	4,280,589 (34.0)
	Terraced	2,647,830 (23.2)	3,045,169 (24.2)

	Flat (purpose built)	927,016 (8.1)	1,311,511 (10.4)
	Flat (converted)	234,296 (2.1)	341,713 (2.7)
	Flat (commercial building)	66,691 (0.6)	79,581 (0.6)
	Other	36,999 (0.3)	40,651 (0.3)
	No code	135,231 (1.2)	145,004 (1.2)
Household size (%)	1-2	7,277,431 (63.6)	7,908,032 (62.8)
	3-4	3,576,237 (31.3)	3,985,460 (31.7)
	5-6	403,703 (3.5)	495,756 (3.9)
	7+	43,368 (0.4)	56,885 (0.5)
	Missing	135,231 (1.9)	145,004 (1.15)
Multigenerational household (%)	No	10,054,231 (87.9)	10,963,173 (87.1)
	Yes	1,246,508 (10.9)	1,482,960 (11.8)
	No code	135,231 (1.2)	145,004 (1.2)
Household with children (%)	Yes	2,232,421 (19.5)	2,497,492 (19.8)
Overcrowded (%)	Yes	648,996 (5.7)	875,471 (6.95)
Key worker type (%)	Education and childcare	708,038 (6.19)	811,577 (6.5)
	Food and necessary goods	92,675 (0.8)	76,209 (0.6)
	Health and social care	856,170 (7.5)	952,736 (7.6)
	Key public services	173,130 (1.5)	205,401 (1.6)
	National and Local Government	93,822 (0.8)	112,471 (0.9)
	Not keyworker	9,061,425 (79.2)	9,947,345 (79.0)
	Public safety and national security	163,166 (1.4)	168,198 (1.3)
	Transport	139,617 (1.2)	142,941 (1.2)
	Utilities and communication	147,927 (1.3)	174,259 (1.4)
Key worker in household (%)	No code	135,231 (1.2)	145,004 (1.2)
	No	7,411,170 (64.8)	8,142,092 (64.7)
	Yes	3,889,569 (34.0)	4,304,041 (34.18)
Proximity to others* (mean [SD])		58.5 (19.4)	58.0 (20.0)
Exposure to disease* (mean [SD])		19.7 (21.1)	19.2 (21.2)
Chronic Kidney disease (%)	None, CKD 1-2	11,267,353 (98.5)	12,259,694 (97.4)
	CKD 3	146,007 (1.3)	287,774 (2.3)
	CKD 4	14,677 (0.1)	30,230 (0.2)
	CKD 5	7,933 (0.1)	13,439 (0.1)

Learning disability (%)	No	11,307,924 (98.9)	12,439,251 (98.8)
	Learning disability	125,410 (1.1)	147,678 (1.2)
	Down's syndrome	2,636 (0.0)	4,208 (0.0)
Cancer and immunosuppression (%)	Blood cancer	145,712 (1.27)	166,064 (1.3)
	Respiratory cancer	4,309 (0.0)	5,163 (0.0)
	Taking immunosuppressants	3,377 (0.0)	3,501 (0.0)
	Taking anti-leukotriene or long acting beta2-agonists	941,990 (8.2)	1,103,917 (8.8)
	Taking oral steroids in the last 6 months	179,500 (1.6)	202,123 (1.6)
Other comorbidities (%)	Cerebral Palsy	1,574 (0.0)	1,939 (0.0)
	Asthma	1,227,304 (10.7)	1,534,364 (12.2)
	Atrial Fibrillation	497,090 (4.4)	590,033 (4.69)
	Coronary heart disease	703,165 (6.2)	839,975 (6.7)
	COPD	479,764 (4.2)	567,190 (4.5)
	Cystic fibrosis or bronchiectasis or alveolitis	155,800 (1.4)	195,170 (1.6)
	Dementia	194,006 (1.7)	220,041 (1.8)
	Diabetes	1,268,015 (11.1)	1,584,898 (12.6)
	Epilepsy	121,092 (1.1)	151,120 (1.2)
	Heart failure	248,579 (2.2)	290,160 (2.3)
	Liver cirrhosis	35,152 (0.3)	43,515 (0.4)
	Neurological disease	11,339 (0.1)	13,601 (0.1)
	Parkinson's disease	51,350 (0.5)	61,893 (0.5)
	Peripheral vascular disease	137,143 (1.2)	164,803 (1.3)
	Fracture of hip, wrist, spine or humerus	10,999 (0.1)	18,031 (0.1)
	Rheumatoid arthritis or SLE	133,379 (1.2)	165,112 (1.3)
	Severe mental illness	2,220,379 (19.4)	2,413,101 (19.2)
	Solid organ transplant	1,296 (0.0)	1,527 (0.0)
	Stroke or TIA	407,668 (3.6)	483,295 (3.8)
	Thrombosis or pulmonary embolus	2,458 (0.0)	3,172 (0.0)

Data as number (column %) or mean (SD).

* = score from 0 (no exposure) to 100 (maximum exposure)

Supplementary Table 3: Statistics showing predictive discrimination for missing data.

**Predictor Variable (outcome missing yes/no)**	**Pseudo R^2**	**AUROC**
Region	0.2086	0.7827
+ Ethnicity and all included covariates	0.2133	0.7928
+outcome (COVID-19 mortality)	0.2133*	0.7928*

* = The outcome does not predict missingness, conditioned on covariates

Supplementary Figure 1: BMI equivalency values for Class II and Class III obesity thresholds across ethnic groups.

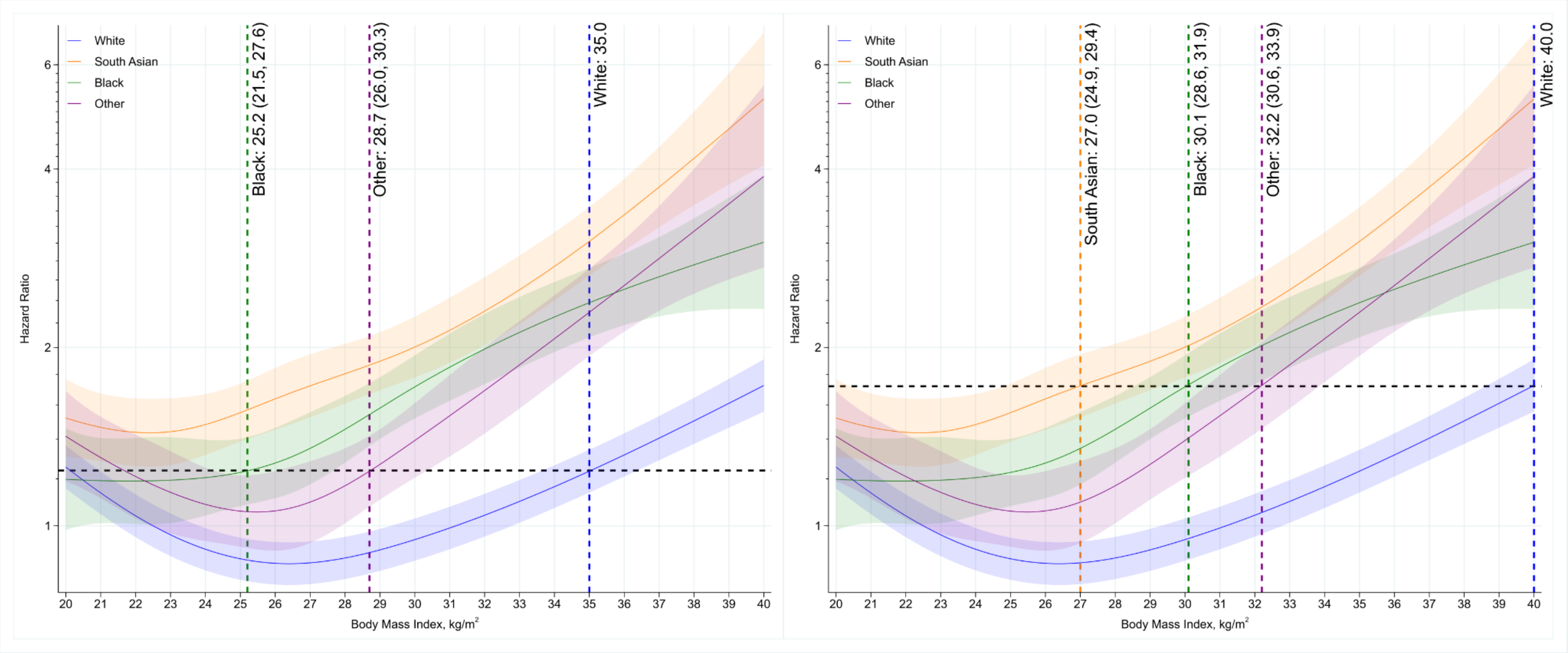

Shaded area as 95% CI

Panel A (left): BMI values in ethnic minority groups for equivalent risk to white ethnicities at a BMI of 35 kg/m^2 [HR = 1.24 (1.34, 1.14)].

Panel B (right): BMI values in ethnic minority groups for equivalent risk to white ethnicities at a BMI of 40 kg/m^2 [HR = 1.73 (1.59, 1.91)]. Model adjusted for: age, sex and demographic variables

Data adjusted for region, population density, urban/rural classification, deprivation (area and household), social grade, qualification, household size, household composition (multigenerational, with children), key worker status and type, occupational exposure to disease, occupational exposure to others.

Supplementary Figure 2: Association of BMI with COVID-19 mortality by ethnicity, adjusted for clinical factors.

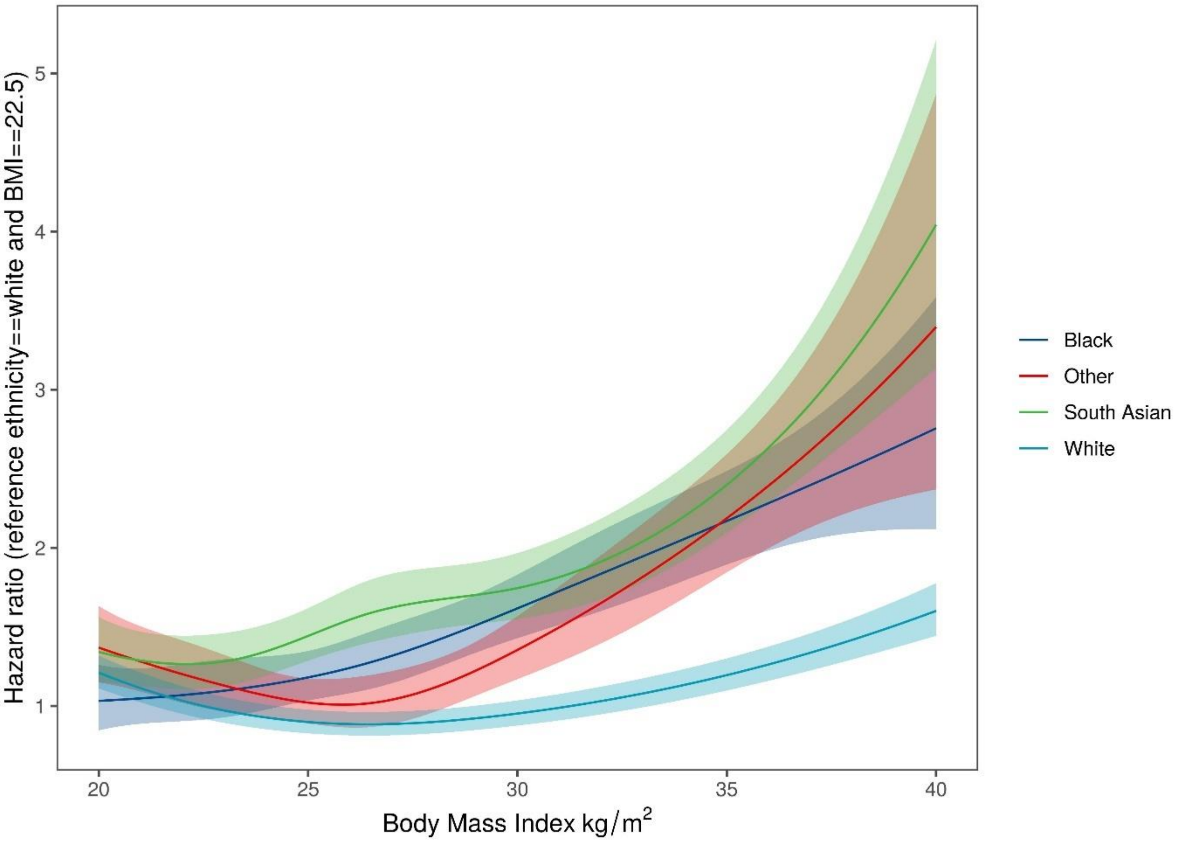

Association of body mass index (BMI) with COVID-19 mortality in white, black, South Asian and 'other' ethnicity. Hazard ratio (HR) for COVID-19 mortality with BMI stratified by ethnic groups.

Reference (HR 1) were placed at BMI of 22.5 kg/m^2 for white individuals. HR (lines) and confidence intervals (CI; areas) are plotted across continuous BMI values. Shaded area as 95% CI.

Analysis adjusted for: age, sex, region, sociodemographic and clinical factors (Model 2 – detailed in Table 1, main text).

Supplementary Figure 3. Association of BMI with COVID-19 mortality by ethnicity in women.

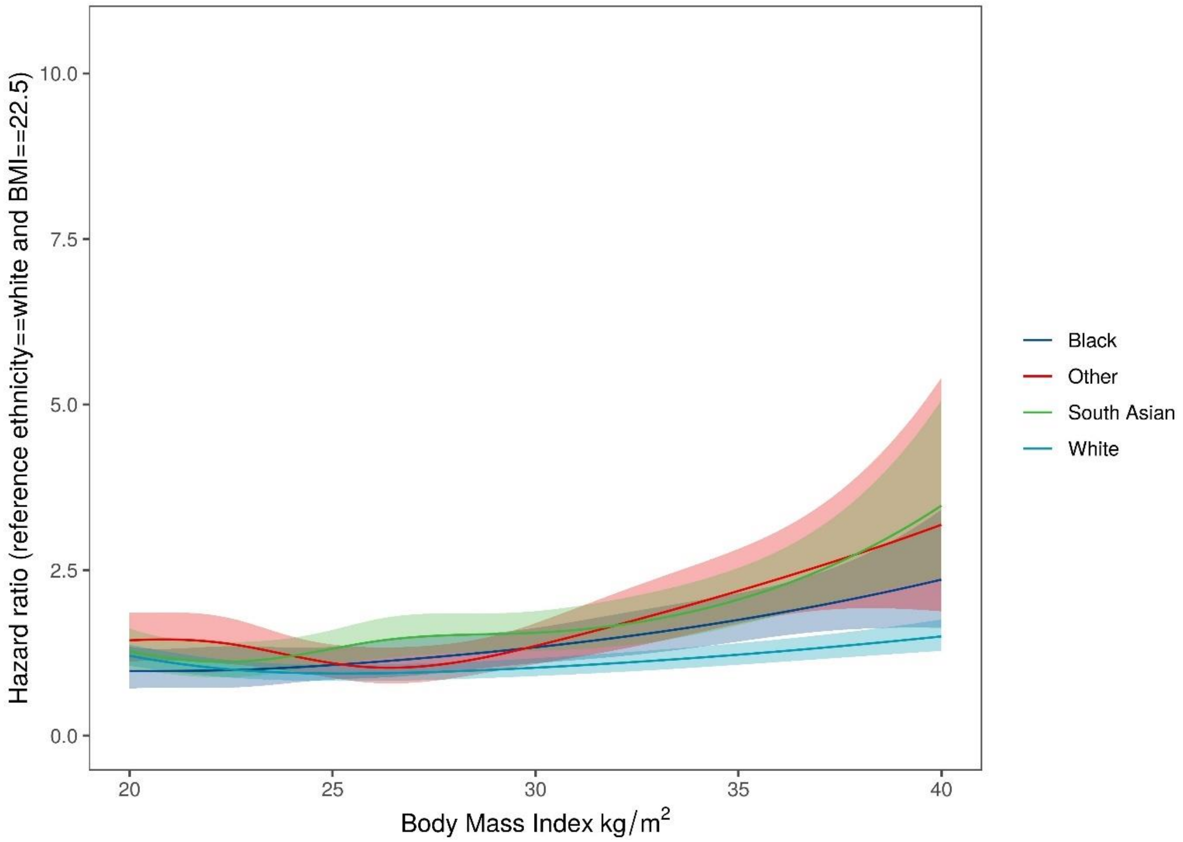

Association of body mass index (BMI) with COVID-19 mortality in white, black, South Asian and 'other' ethnicity women. Hazard ratio (HR) for COVID-19 mortality with BMI stratified by ethnic groups.

Reference (HR 1) were placed at BMI of 22.5 kg/m^2 for white individuals. HR (lines) and confidence intervals (CI; areas) are plotted across continuous BMI values. Shaded area as 95% CI.

Analysis adjusted for: age, region and sociodemographic factors (Model 1 – detailed in Table 1, main text).

Supplementary Figure 4: Association of BMI with COVID-19 mortality by ethnicity in men.

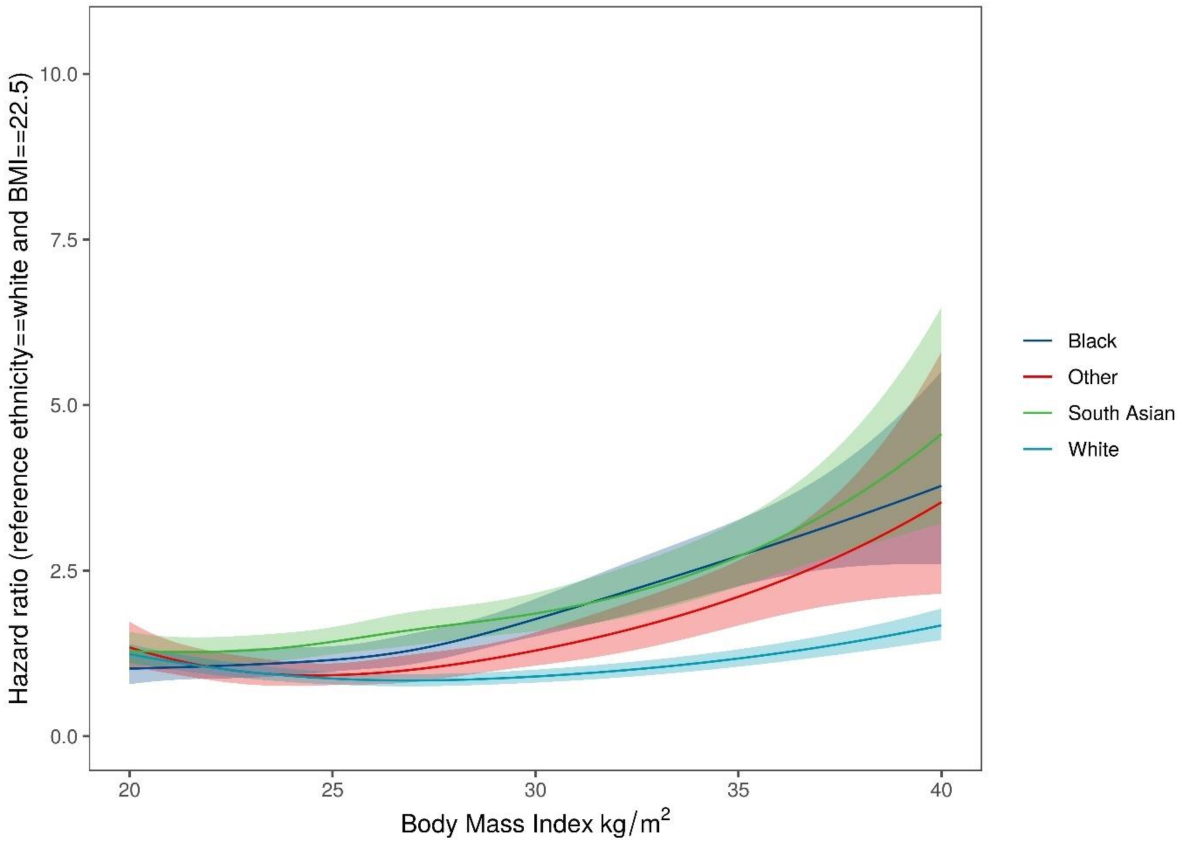

Association of body mass index (BMI) with COVID-19 mortality in white, black, South Asian and 'other' ethnicity men. Hazard ratio (HR) for COVID-19 mortality with BMI stratified by ethnic groups.

Reference (HR 1) were placed at BMI of 22.5 kg/m2 for white individuals. HR (lines) and confidence intervals (CI; areas) are plotted across continuous BMI values. Shaded area as 95% CI.

Analysis adjusted for: age, region and sociodemographic factors (Model 1 – detailed in Table 1, main text).

Supplementary Figure 5: Association of BMI with COVID-19 mortality by ethnicity: individuals 70 years or older.

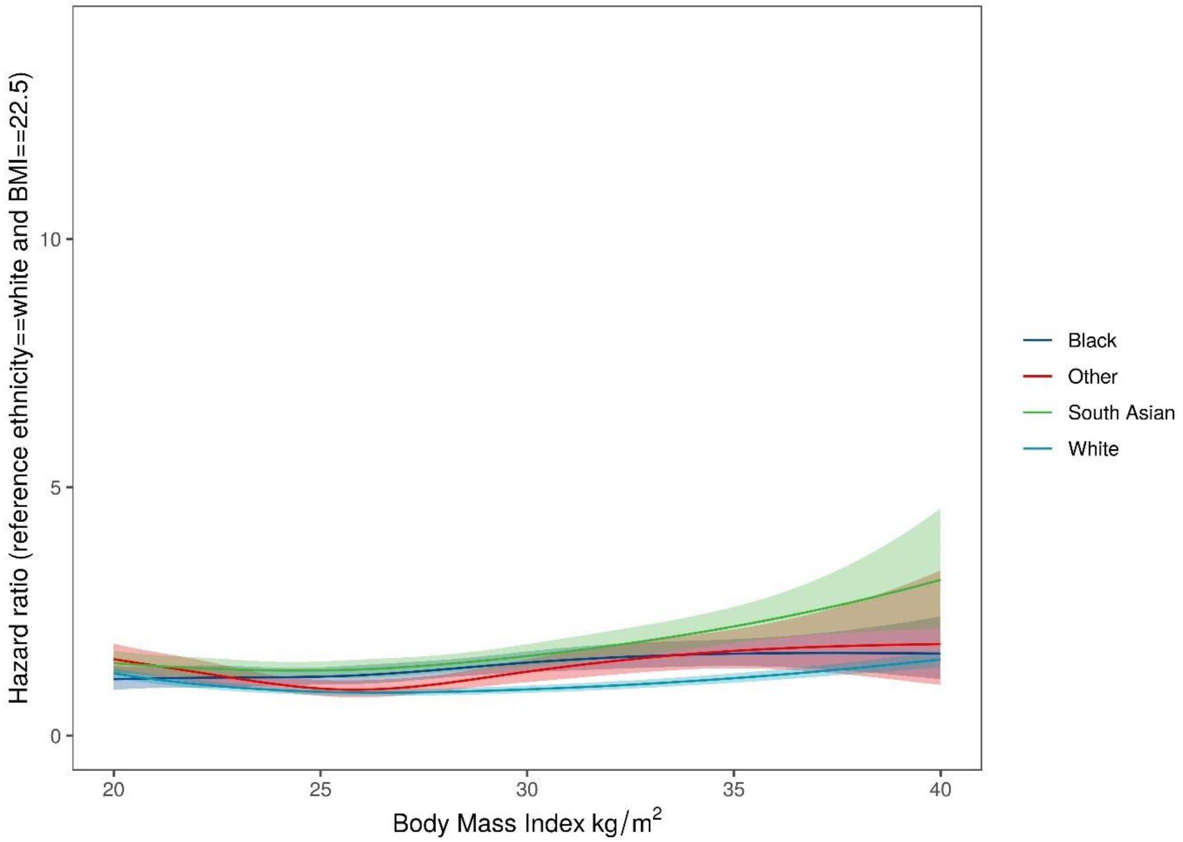

Association of body mass index (BMI) with COVID-19 mortality in white, black, South Asian and 'other' ethnicity individuals. Hazard ratio (HR) for COVID-19 mortality with BMI stratified by ethnic groups.

Reference (HR 1) were placed at BMI of 22.5 kg/m^2 for white individuals. HR (lines) and confidence intervals (CI; areas) are plotted across continuous BMI values. Shaded area as 95% CI.

Analysis adjusted for: age, sex, region and sociodemographic factors (Model 1 – detailed in Table 1, main text).

Supplementary Figure 6: Association of BMI with COVID-19 by ethnicity: individuals younger than 70 years.

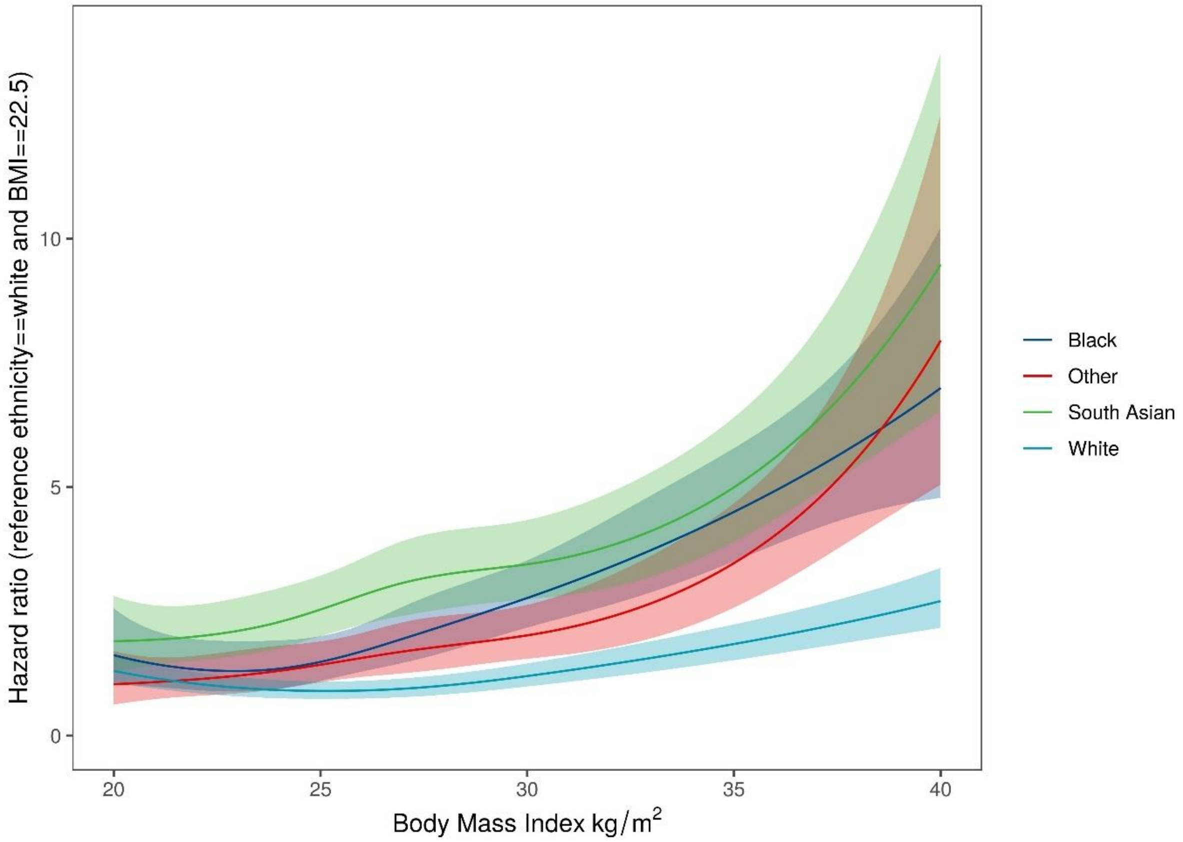

Association of body mass index (BMI) with COVID-19 mortality in white, black, South Asian and ‘other’ ethnicity individuals. Hazard ratio (HR) for COVID-19 mortality with BMI stratified by ethnic groups.

Reference (HR 1) were placed at BMI of 22.5 kg/m^2 for white individuals. HR (lines) and confidence intervals (CI; areas) are plotted across continuous BMI values. Shaded area as 95% CI.

Analysis adjusted for: age, sex, region and sociodemographic factors (Model 1 – detailed in Table 1, main text).

Supplementary Figure 7: Association of BMI with COVID-19 mortality across ten ethnic categories.

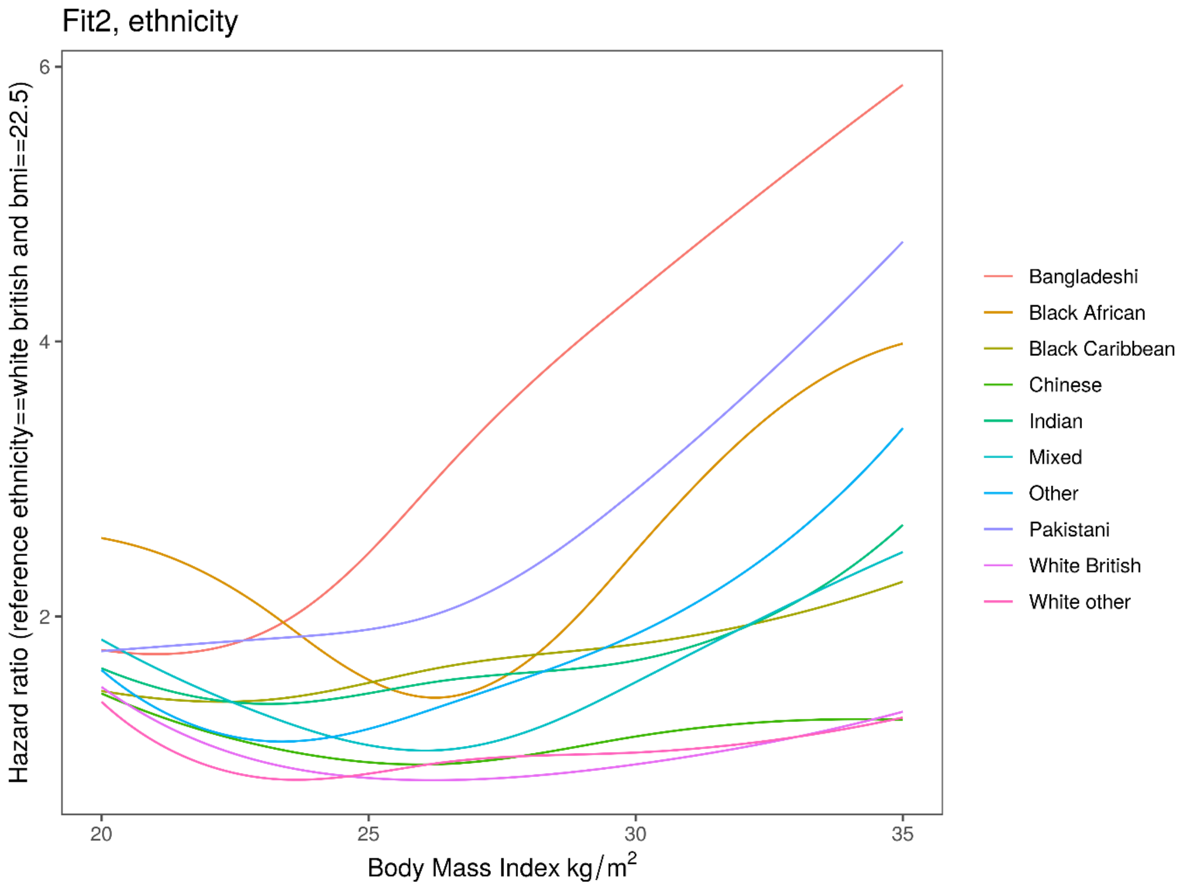

Reference (HR 1) were placed at BMI of 22.5 kg/m^2 for white individuals. Analysis adjusted for: age, sex, region and sociodemographic factors (Model 1: detailed in Table 1, main text).

Supplementary Figure 8: Flow diagram of cohort.

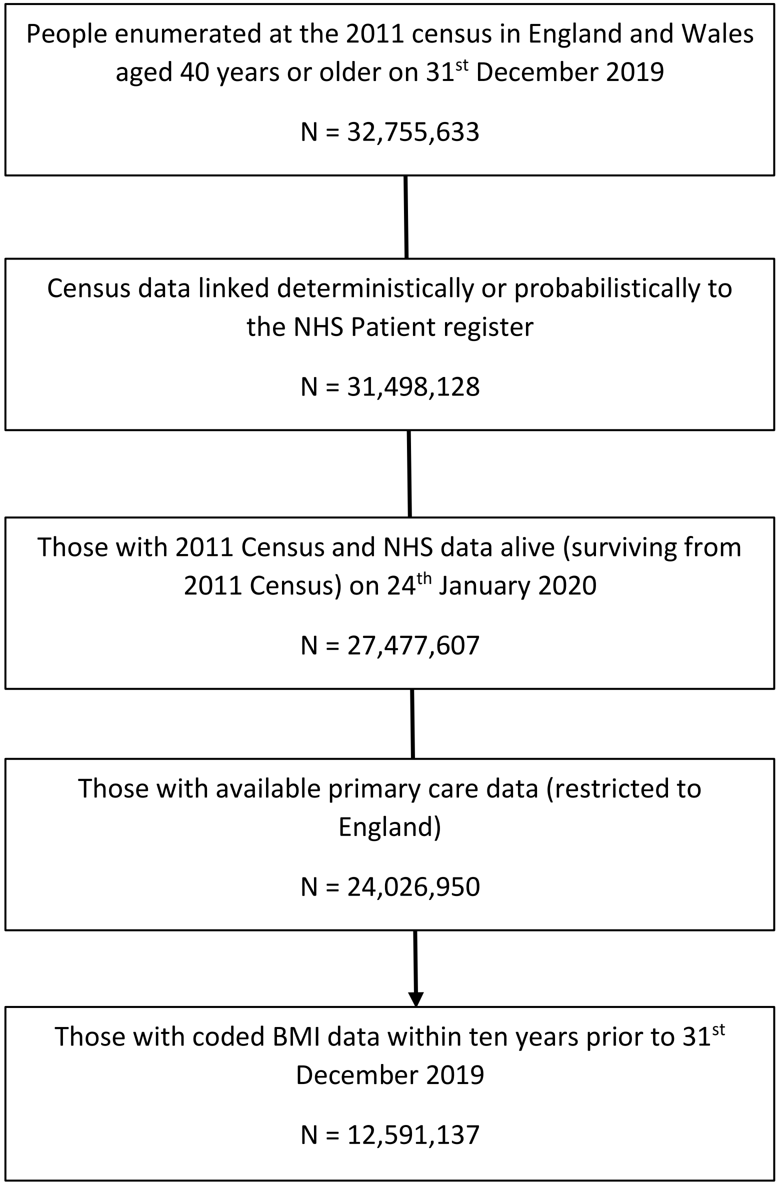

Supplementary Figure 9: Percentage of missing BMI data in each ethnic group by region.

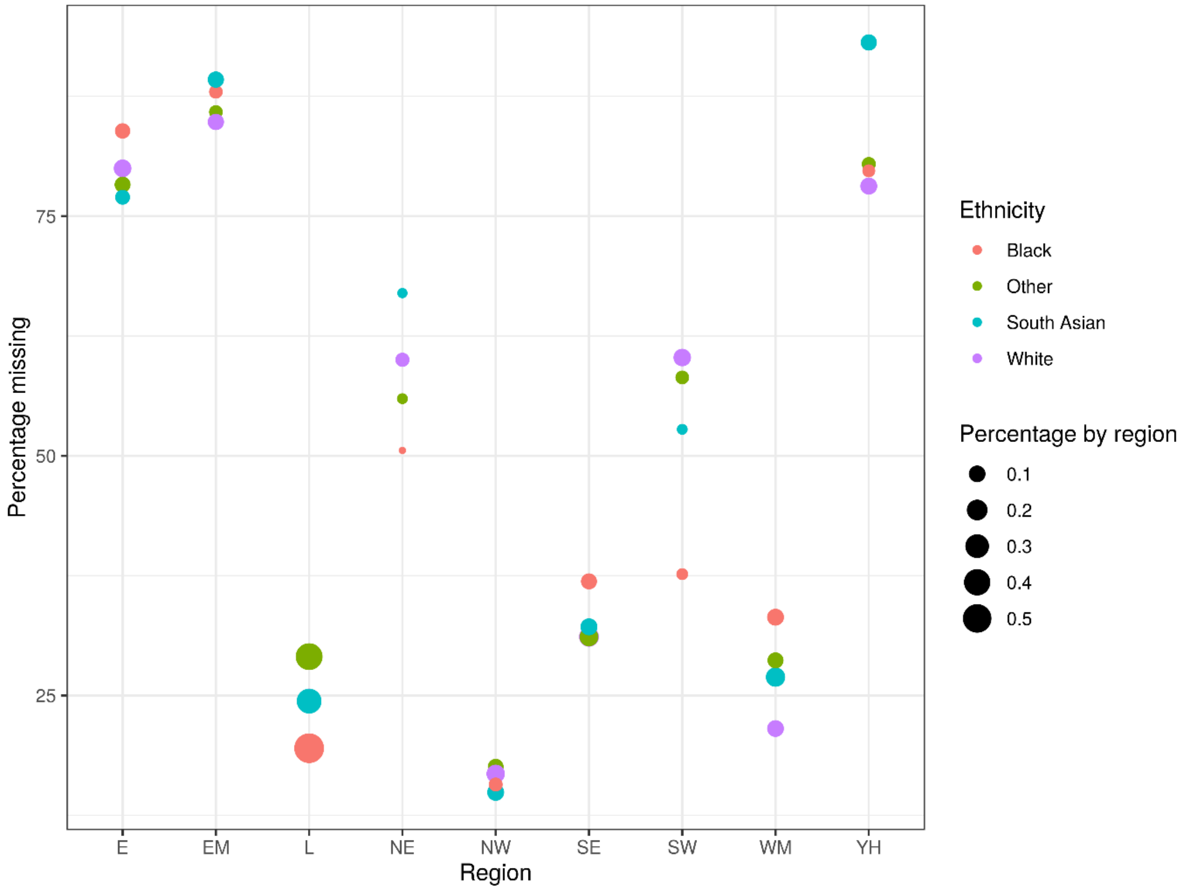

E: East of England; EM: East Midlands; L: London; NE: North East; NW: North West; SE: South East; SW: South West; WM: West Midlands; YH: Yorkshire and Humber.

The size of dot is proportional to the number of people within that specific ethnic group across all nine regions (proportion = 1).
