# Supplementary material for: A population-based cohort study of obesity, ethnicity and COVID-19 mortality in 12.6 million adults in England
Source: Nat Commun. 2022 Feb 2;13:624. doi: 10.1038/s41467-022-28248-1 (PMC8810846; doi:10.1038/s41467-022-28248-1)
Supplement: Supplementary file 3 — Description of Additional Supplementary Files [file 41467_2022_28248_MOESM3_ESM.pdf]

Description of Additional Supplementary Files

File Name: Supplementary Data 1
Description: Descriptive profile of continuous factors stratified by ethnicity and BMI category. Data shown as mean (standard deviation).

File Name: Supplementary Data 2
Description: Descriptive profile of categorical factors stratified by ethnicity and BMI category. Data shown as count (percentage).
