# Supplementary material for: A population-based cohort study of obesity, ethnicity and COVID-19 mortality in 12.6 million adults in England
Source: Nat Commun. 2022 Feb 2;13:624. doi: 10.1038/s41467-022-28248-1 (PMC8810846; doi:10.1038/s41467-022-28248-1)
Supplement: Supplementary file 6 — Reporting Summary [file 41467_2022_28248_MOESM6_ESM.pdf]

nature portfolio

Corresponding author(s): Prof Tom Yates

Last updated by author(s): Nov 22, 2021

Reporting Summary

Nature Portfolio wishes to improve the reproducibility of the work that we publish. This form provides structure for consistency and transparency in reporting. For further information on Nature Portfolio policies, see our Editorial Policies and the Editorial Policy Checklist.

Statistics

For all statistical analyses, confirm that the following items are present in the figure legend, table legend, main text, or Methods section.

n/a	Confirmed	
☒	☐	The exact sample size (*n*) for each experimental group/condition, given as a discrete number and unit of measurement
☐	☒	A statement on whether measurements were taken from distinct samples or whether the same sample was measured repeatedly
☐	☒	The statistical test(s) used AND whether they are one- or two-sided<br>*Only common tests should be described solely by name; describe more complex techniques in the Methods section.*
☐	☒	A description of all covariates tested
☐	☒	A description of any assumptions or corrections, such as tests of normality and adjustment for multiple comparisons
☐	☒	A full description of the statistical parameters including central tendency (e.g. means) or other basic estimates (e.g. regression coefficient) AND variation (e.g. standard deviation) or associated estimates of uncertainty (e.g. confidence intervals)
☐	☒	For null hypothesis testing, the test statistic (e.g. *F*, *t*, *r*) with confidence intervals, effect sizes, degrees of freedom and *P* value noted<br>*Give P values as exact values whenever suitable.*
☐	☐	For Bayesian analysis, information on the choice of priors and Markov chain Monte Carlo settings
☐	☐	For hierarchical and complex designs, identification of the appropriate level for tests and full reporting of outcomes
☒	☐	Estimates of effect sizes (e.g. Cohen's *d*, Pearson's *r*), indicating how they were calculated

Our web collection on statistics for biologists contains articles on many of the points above.

Software and code

Policy information about availability of computer code

Data collection	The statistical codes used for the described analysis can be found online with the following DOI
Data analysis	Data analysis was undertaken in R version 3.5. Open source packages used were sparklyR, survival, rms

For manuscripts utilizing custom algorithms or software that are central to the research but not yet described in published literature, software must be made available to editors and reviewers. We strongly encourage code deposition in a community repository (e.g. GitHub). See the Nature Portfolio guidelines for submitting code & software for further information.

Data

Policy information about availability of data

All manuscripts must include a data availability statement. This statement should provide the following information, where applicable:

- Accession codes, unique identifiers, or web links for publicly available datasets
- A description of any restrictions on data availability
- For clinical datasets or third party data, please ensure that the statement adheres to our policy

Analysed data are controlled by the Office of National Statistics, UK. Technical details of the Public Health Research Database (PHRD) incorporating the 2011 Census data for England and Wales, linked to Mortality Data, Hospital Episode Statistics (HES) data, and GP Extraction Service (GPES) data for Pandemic Planning and Research Data can be found through the Health Data Research UK Innovation Gateway https://web.www.healthdatagateway.org/dataset/a325f33e-bac8-49af-896f-1e025941dae8 Given the sensitive nature of the data, organisations and individuals will need to demonstrate they meet strict data security and information governance standards. The application form can be accessed and completed through Health Data Research UK Innovation Gateway https://web.www.healthdatagateway.org/dataset/a325f33e-bac8-49af-896f-1e025941dae8

Field-specific reporting

Please select the one below that is the best fit for your research. If you are not sure, read the appropriate sections before making your selection.

☒ Life sciences ☐ Behavioural & social sciences ☐ Ecological, evolutionary & environmental sciences

For a reference copy of the document with all sections, see nature.com/documents/nr-reporting-summary-flat.pdf

Life sciences study design

All studies must disclose on these points even when the disclosure is negative.

Sample size	Assuming a cumulative incidence of 8 COVID mortality events per 10,000 people and a standard deviation of BMI of 6 kg/m2 [both from doi: 10.1016/S2213-8587(21)00089-9], 8.7 million subjects were required to detect an association between BMI and COVID mortality with at least a hazard ratio of 1.01 per 1-unit increase in BMI, with a model Rsquared of 0.4 [worst case scenario], alpha of 0.01, and power of 0.9.
Data exclusions	Those without the exposure of interest (BMI) were excluded a priori.
Replication	This analysis reports associations using population level data from England during the first year of the pandemic. Therefore replication was not applicable or possible.
Randomization	As this was a cohort study investigating associations with COVID-19 mortality, randomization was not applicable
Blinding	As this was a population level cohort study investigating associations with COVID-19 mortality, blinding was not applicable

Reporting for specific materials, systems and methods

We require information from authors about some types of materials, experimental systems and methods used in many studies. Here, indicate whether each material, system or method listed is relevant to your study. If you are not sure if a list item applies to your research, read the appropriate section before selecting a response.

Materials & experimental systems

n/a	Involved in the study	
☒	☐	Antibodies
☒	☐	Eukaryotic cell lines
☒	☐	Palaeontology and archaeology
☒	☐	Animals and other organisms
☐	☒	Human research participants
☒	☐	Clinical data
☒	☐	Dual use research of concern

Methods

n/a	Involved in the study	
☒	☐	ChIP-seq
☒	☐	Flow cytometry
☒	☐	MRI-based neuroimaging

Human research participants

Policy information about studies involving human research participants

Population characteristics	This analysis included 11,074,708 (53.6% women, 61.9 [±13.4] years) white, 416,542 (57.3% women, 56.4 [±11.7] years) black, 621,691 (51.0% women, 55.7 [±12.4] years) South Asian and 478,196 (54.9% women, 55.3 [±11.6] years) other ethnic groups with linked BMI data. The full descriptive profile of the cohort, including covariate details, is provided in Table 1 in the manuscript.
Recruitment	This analysis used national census data and routine clinical records, therefore patient recruitment was not required. Potential for bias was introduced by missing BMI data from clinical records. As BMI is likely to be missing not at random and influenced by many factors, not all of which were captured in this analysis, multiple imputation of missing data was not attempted. The potential impact of missing data is detailed in the Discussion section of the manuscript: p14-15
Ethics oversight	Ethics for this project and analysis was provided by the University of Leicester, UK

Note that full information on the approval of the study protocol must also be provided in the manuscript.
